# Solar radiation and building integrated photovoltaic power generation datasets at zero emission building laboratory, Trondheim

**DOI:** 10.1016/j.dib.2026.113014

**Published:** 2026-06-23

**Authors:** Mulu Bayray Kahsay, Natasa Nord

**Affiliations:** Department of Energy and Process Engineering, NTNU, Kolbjørn Hejes v 1B, 7034, Trondheim, Norway

**Keywords:** Building energy, Facades and roof, Measurement datasets, High latitude

## Abstract

This paper presents a high-resolution one-hour time interval, three-years datasets of solar irradiance and corresponding photovoltaic power generation collected from a Zero Emission Building (ZEB) located in high latitude climate at Trondheim, Norway. The datasets (one dataset on solar irradiance and a second dataset on PV power generation) include measurements from facades oriented in the North, East, West, South as well as Pergola shade (60° tilt) and Roof top (30° tilt). The datasets capture real-world operating conditions, including seasonal variability, low solar angles, and the influence of snow and cloud cover as well as building shading providing a comprehensive basis for evaluating PV system performance under challenging climate conditions. By combining measured solar radiation with synchronized PV output, the datasets enable detailed analysis of system efficiency, performance ratios, and temporal dynamics across multiple scales.

The datasets support statistical assessment of inter-annual variability and offer insights into potential performance degradation and system reliability. The datasets are useful for validating and calibrating solar energy models, as well as for developing and benchmarking forecasting methods. It is expected to support further research on a wide range of applications, and policy analysis aimed at advancing sustainable and zero-emission buildings.

Specifications Table

**Table:** 

Subject	Engineering & Materials science
Specific subject area	Solar radiation measurement from pyranometers at different orientations and photovoltaic electric power generation from arrays installed on facades and roof.
Type of data	Table in the form of Excel data sheet. Raw data filtered to qualify for missing and erroneous data.
Data collection	Solar radiation measurements were carried out employing DeltaOHM model LPPYRA03S, Spectrally Flat Class C Pyranometers. Ambient temperature measurement data was from the weather station installed in the building with PT100 temperature sensor. PV electric power generation data was calculated from current and voltage measurements from three inverters and respective MPPT controllers for each string in the PV array. Inverters are from Sungrow, models CX110, CX50 and KTL-M. [https://zeblab.sintef.no/wiki/Data_Recording] Datasets for the years 2023, 2024 and 2025 were downloaded from ZEB Lab Influxdb database at one hour interval. Raw data has been filtered and re-organized into annual Tables (2023, 2024, and 2025) for ease of data analysis. [https://zeblab.sintef.no:8086/orgs/66dfdb36dd042ddc/data-explorer] Graphical displays of the ZEB solar radiation and PV power generation information are publicly available at the web page of the ZEB lab. [https://zeblab.sintef.no/grafana/dashboards]
Data source location	ZEB Lab Trondheim, Norway (coordinate: 63° 25′ N, 10° 27′ E) (www.zeblab.no)
Data accessibility	Repository name: Mendeley Data identification number: doi: 10.17632/g86rtgzvkh.2 Direct URL to data: The datasets are available in the Mendeley open source with a title “ZEB Lab Measured Solar Radiation and PV Power Datasets” [1] https://data.mendeley.com/datasets/g86rtgzvkh/2
Related research article	Performance of Building Integrated Photovoltaic (BIPV) in Zero Emission Building (ZEB) at High Latitudes: the case of ZEB Lab, Trondheim, Norway Manuscript accompanying submission. [2]

## 1 Value of the Data

• The datasets comprising three years of measured solar radiation and corresponding photovoltaic generation from a zero-emission building represent a valuable contribution to the application of solar energy in buildings. Unlike simulated datasets, it captures real-world variability influenced by weather conditions, including seasonal changes, cloud covers, snow effects, and shading from nearby buildings.
• The simultaneous availability of irradiance and PV output at facades and roof orientations enables detailed performance analysis such as efficiency estimation, performance ratio evaluation and system diagnosis.
• The multi-year duration enhances statistical reliability and allows investigation of inter-annual variability and potential system degradation. Such datasets are especially useful for validating and calibrating energy models, improving forecasting methods, and supporting research on building integrated energy systems.
• Considering the limited availability of long-term, high-quality measurement data from high latitude climates, the dataset can support a wide range of applications, including PV system design, energy system optimization, and policy development in sustainable buildings.

## 2 Background

The compilation of the solar radiation and photovoltaic power generation datasets is based on the ambitions of the zero emission buildings research, which aims to enable buildings that achieve net zero greenhouse gas emissions over their lifetime. The ambitions and design concepts of the ZEB laboratory in Trondheim, Norway, have been reported in literature [3,4]. In high latitudes such as Norway, where solar resources are highly variable and strongly seasonal, there is a critical need for reliable, long term empirical data to support the design and operation of energy systems that can meet theses ambitious targets. The motivation behind these datasets arises from the lack of detailed, synchronized measurements of both solar irradiance and actual PV performance under real climatic conditions. While simulations tools are widely used in building design, their accuracy depends heavily on the quality of input data and validation against measured performance. The datasets with continuous data for three years will contribute to addressing this gap. The datasets capture not only typical conditions but also extreme and transitional periods, such as low winter irradiance, snow, and cloud coverage as well as shading from buildings.

## 3 Data Description

There are two datasets in the compilation: i) solar irradiance and ambient temperature dataset, and ii) PV power generation dataset. Both datasets cover three years 2023, 2024 and 2025. The datasets are compiled as Excel sheet Tables as described below.

### 3.1 Solar irradiance and temperature dataset

The dataset contains three Excel files respectively for each year with 13 columns, where the first row is the data label. The first four columns are Year, Month, Day and Hour, where the Hour runs from 0 to 23. The time interval is one hour. The time stamp is based on the average values of measured data records within the past one hour. For example, the time stamp 10:00 represents the average value of the measured records during the period 09:00 to 10:00. Table 1 describes the remaining columns.

**Table 1:** Description of the data columns in the files.

Column No.	Label	Description/Unit	Orientation		Remark
			Tilt	Azimuth	
1 −4	Year, Month, Day, Hour				
5	North	Global Tilted Irradiance (GTI), W/m^2^	90°	0°	
6	West		90°	240°	The pyranometer is aligned with the facade which is not perfectly on the West orientation
7	East		90°	90°	
8	South		90°	180°	
9	Pergola_outwards		60°	180°	On the outside of the Pergola
10	Pergola_inwards		60°	180°	In the inside of the Pergola
11	Roof_Angle		30°	180°	
12	Roof_Horizontal	Global Horizontal Irradiance (GHI), W/m^2^	0	-	Pyranometer at roof top but installed horizontally
13	Temperature	Ambient Temperature, °C			

### 3.2 PV power generation dataset and building electricity use

Similarly, this dataset contains three Excel files respectively for the three years with 11 columns where the first four columns are Year, Month, Day and Hour. Table 2 describes the orientation of the PV arrays where the PV power generation data was collected. Included in Table 2 is the building electricity use as 11th column in the dataset. The hourly electricity use of the building was extracted separately from the energy meter in the building and included here for any comparative analysis between the energy generated and utilized in the building.

**Table 2:** Description of the location and orientation of the PV arrays.

Column No.	Label	Description/Unit	Orientation		Remark
			Tilt	Azimuth	
1 – 4	Year, Month, Day, Hour				
5	North	PV Power Generated, Wh	90°	0°	
6	West		90°	240°	The facade is not perfectly on the West orientation
7	East		90°	90°	
8	South		90°	180°	
9	Pergola		60°	180°	
10	Roof_Angle		30°	180°	
11	Electric use	Power in W			From energy meter of the building

## 4 Experimental Design, Materials and Methods

**Fig. 1:**
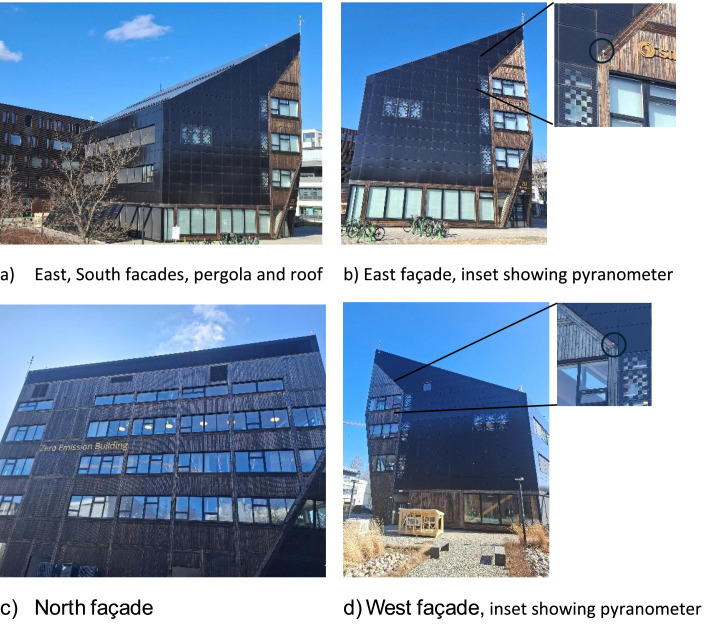
The ZEB Lab, pictures showing the facades, pergola and roof. [Photos: Mulu Bayray Kahsay].

ZEB Lab is a four-story research building owned by NTNU and SINTEF used as an office building (picture shown in Fig. 1). The design, planning and construction process has been reported in [3,4]. The building has a total area of 2000 m^2^, primarily used for offices with additional rooms for educational space for lectures and meetings [4]. The BIPV envelope includes the roof, pergola and the facades as described in Table 2. The BIPV in the roof has been mounted with integrated frame system attached with brackets to the roof. He BIPV on the facades are mounted as rain screen cladding with vertical and horizontal fastening system. The details of the BIPV installation is reported in [5]. The building energy related data are available for the public in Grafana dashboards [6] and also available in the ZEB Lab Influxdb database [7]. The ZEB Lab has measurement and monitoring instruments installed over the facades and the roof. Manni et al., in their studies on solar energy digitalization [8], have described the locations of the installation of the pyranometers. The datasets compiled here are extracted from the wider measurement and monitoring instrumentation of the ZEB Lab. The solar radiation dataset is from eight pyranometers installed at the facades and the roof. The PV power production dataset has been also extracted from the current and voltage measurement at the PV array inverters and MPPT controllers. The pyranometers used are calibrated with the reference sample at World Radiation Center (WRC) [6]. Table 3 shows the description of the instruments and the uncertainty of measurement.

**Table 3:** Description of measurement instruments.

Instrument	Model	Classification	Uncertainty
Pyranometer	DelatOHM LPPYRA03S	Spectrally Flat Class C	Directional 2 % Spectral 2 % Non-linearity 1.5 % Equivalent total uncertainty 3.2 %
Thermometer	PT100		±0.15 °C
Inverter Voltage and Current	Sungrow CX110, CX50 and KTL-M		±0.5 % to ±1.0 % Equivalent standard uncertainty for power ±0.7 % to ±1.4 %

The data was accessed from the ZEB Lab Influxdb explorer (with permission from NTNU/SINTEF). The following query procedure was followed to access the data. Table 4 shows the query filter values employed to extract solar radiation and temperature data from the seven pyranometers (PYRA1 to 7) and weather station (WS2: a Pyranometer and a Temperature sensor).

**Table 4:** Filter values to extract solar radiation and temperature.

*Item*	*Filter value*
Bucket	ZEB
Measurement Tag	570.001
Position	North, West, East, South, Pergola_inwards, Pergola_outwards, Roof_Angle, Roof
Component	ZEB_PYRA1, ZEB_PYRA2, ZEB_PYRA3, ZEB_PYRA4, ZEB_PYRA5, ZEB_PYRA6, ZEB_PYRA7, ZEB_WS2
Field	Solar Radiation (W/m^2^), Temperature (°C)

The installation of the PV modules at the ZEB Lab are organized into three arrays and sixteen strings with MMPT controller [9]. The description of the arrays, strings, location and installed power are shown in Table 5. The PV current and voltage data from each string and array was extracted based on the query filter values shown in Table 6. The PV power (W) at each time interval was then found by the product of the current (A) and voltage (V) values.

**Table 5:** Description of the installed PV modules, strings and arrays.

*Array*	*String*	*Location*	*Module power (Wp)*	*No. of Units*	*Installed power (kWp)*	*Module Type*	*Module Efficiency (%)*	*Temperature Coefficient (power)*
Inverter 1 (LQ001)	MPPT1 to MPPT7	Roof	350	280	98.00	Sunpower X1–350 Mono C-Si	21.5	−0.29 %/ °C
	MPPT8 and MPPT9	East	170	144	24.47	Solarlab, Mono C-Si	15.5	NA
Inverter 2 (LQ002)	MPPT1 and MPPT2	Pergola	375 230	21 21	7.875 4.83	Sunpower MAX3–375, Mono C-Si, Solitek Solrif Bifacial Mono C-Si	21.2 13.6	−0.27 %/ °C −0.47 %/ °C
	MPPT3	North	375	30	11.25	Sunpower MAX3–375, Mono C-Si	21.2	−0.27 %/ °C
	MPPT4 and MPPT5	South	170	132	22.36	Solarlab, Mono C-Si	15.5	NA
Inverter 3 (LQ003)	MPPT1 and MPPT2	West	170	73	12.36	Solarlab, Mono C-Si	15.5	NA

NA: Not available.

**Table 6:** Filter values to extract PV current and voltage values from each string.

*Item*	*Filter value*
Bucket	ZEB
Measurement Tag	471.001 and 471.001_1
Area	North, West, East, South, Pergola, Roof
Component	Inverter: LQ001, LQ002, LQ003
Point	Inv1_MPPT1_Current, Inv1_MPPT1_Voltage, Inv1_MPPT2_Current, Inv1_MPPT2_Voltage, etc. for all MPPTs under the three inverters (Inv1, Inv2 and Inv3). Current in Ampere (A) and Voltage in (V)

The extracted raw data was checked for errors and missed records. The missing data is presented under the limitations section. The following are the main remarks regarding data qualification.

• Negative solar radiation data records were observed during nighttime hours. Such data were corrected to zero values in the solar radiation dataset in all cases.
• Current and Voltage data which were flagged as not available (NA) were set to zero and hence the power value was set to zero.

**Fig. 2:**
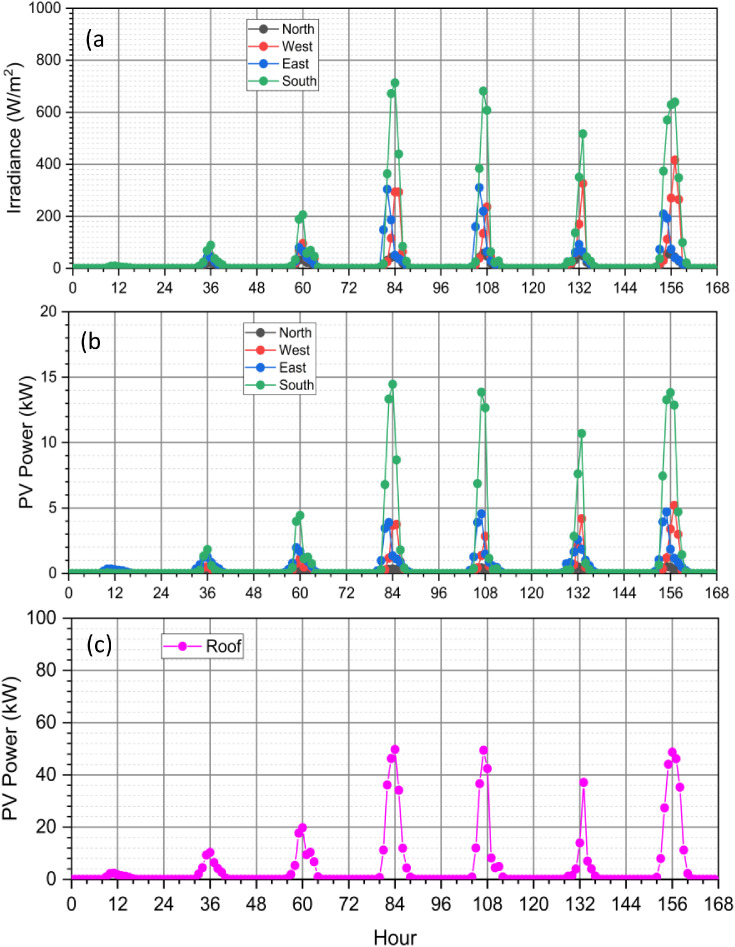
Plots of measured data February 4–10, 2025, a) hourly solar radiation, b) PV power generation at facades, and c) PV power generation at the roof.

**Fig. 3:**
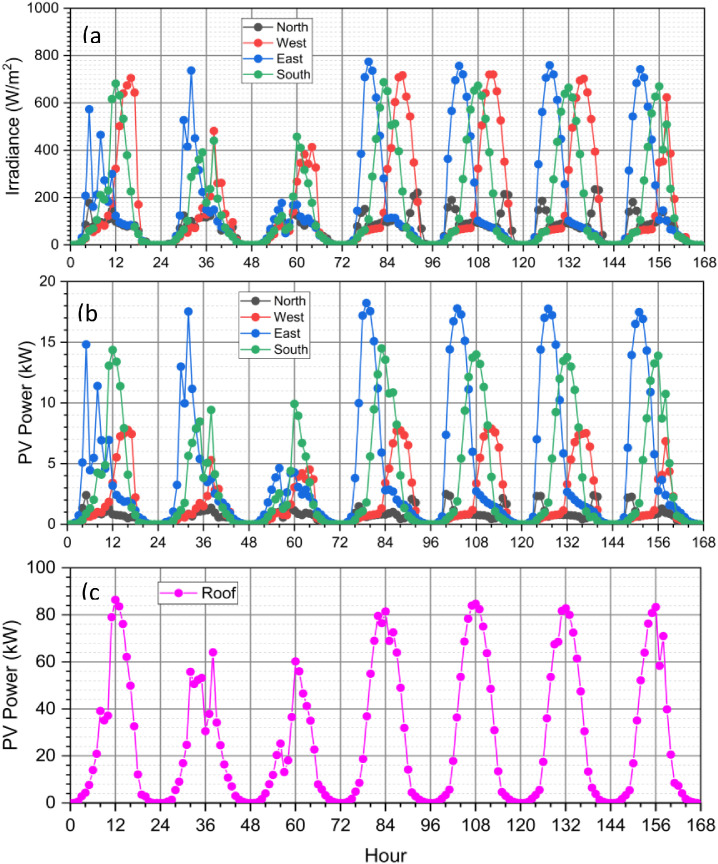
Plots of meausred data July 4–10, 2025 a) hourly solar radiation, b) PV power generation at facades, and c) PV power generation at the roof.

The following figures show plots of the hourly measured data in selected weeks in February and July. Fig. 2 shows solar radiation and PV generation at the different facades and the roof in a winter month. The data indicates first three overcast days with almost no solar radiation and the next four days clear sky conditions. During the clear sky days, the South facade generated close to 15 kW while the roof generated around 50 kW around noon. In contrast Fig. 3 shows the data from the month of July. The peak PV generation at North, West, East and South facades was 3, 8, 14, 18 kW, respectively, while at the roof the peak power was 82 kW around noon.

## Limitations

Overall, the data extracted for the years 2023, 2024, and 2025 were complete with few missing data. The datasets were checked for missing data. No interpolation or data filling was carried out for the missing data.

The solar radiation and temperature dataset has the following missing data:

• Pyranometer data from West facade were not available for 2023 and until March 19, 2024.
• Temperature data were not available 01/01/2023 until 02/01/2023 for 37 h and from 22/08/2023 until 23/08/2023 for 14 h.

Table 7 shows the PV power production missing data. It can be noted that the West façade installation had missing data for the full year 2023 and months January to March in 2024. Table 8 summarizes the annual data completeness statistics where the expected number of data for each parameter was 8760 for 2023 and 2025 while for 2024 the expected number of data was 8784 due to the leap year. The summary indicated that more than 90 % of the expected data were available except in the West facade.

**Table 7:** Missing PV power generation data.

Year	Month	Missing Data	No. of hours
2023	All months	Data for West installation NA	
	Mar	15/03 19:00 to 16/03 12:00	17
		16/03 20:00 to 17/03 05:00	10
	Aug	22/08 18:00 to 23/08 07:00	14
		25/08 07:00 to 09:00	3
		25/08 13:00 to 28/08 07:00	67
		31/08 08:00 to 31/08 22:00	14
	Sep	01/09 00:00 to 02/09 04:00	30
		04/09 14:00 to 05/09 04:00	15
		13/09 20:00 to 16/09 05:00	48
		17/09 05:00 to 18/09 08:00	28
		27/09 16:00 to 30/09 23:00	79
	Oct	01/10 00:00 to 03/10 12:00	61
		08/10 18:00 to 09/10 09:00	14
	Dec	25/12 02:00 to 28/12 11:00	73
2024	Jan	Data for West installation NA	
	Feb	Data for West installation NA	
	Mar	Data for West installation NA	
	Oct	Data for Roof installation NA	
	Dec	11/12 14:00 to 31/12 00:00	20 days
2025	Jan	01/01 00:00 to 31/01 00:00	30 days

**Table 8:** Summary of data completeness statistics.

Parameter	Location	2023		2024		2025	
		Missed data	Available %	Missed data	Available %	Missed data	Available %
Hourly Solar Irradiance	North	0	100	0	100	0	100
	West	8760	0	1896	78.4	0	100
	East	0	100	0	100	0	100
	South	0	100	0	100	0	100
	Pergola	0	100	0	100	0	100
	Roof	0	100	0	100	0	100
Temperature	Roof	51	99.4	0	100	0	100
PV Power Generation	North	473	94.6	480	94.5	720	91.8
	West	8760	0	2662	69.7	720	91.8
	East	473	94.6	480	94.5	720	91.8
	South	473	94.6	480	94.5	720	91.8
	Pergola	473	94.6	480	94.5	720	91.8
	Roof	473	94.6	1224	86.1	720	91.8

## Ethics Statement

The authors have read and followed the ethical guidelines for publication in Data in Brief and confirm that the current work does not involve human subjects, animal experiments, or any data collected from social media platforms.

## CRediT Author Statement

**Mulu Bayray Kahsay:** Conceptualization, Methodology, Data curation, Writing-review and editing; **Natasa Nord:** Conceptualization, Methodology, Supervision, Writing-review and editing.

## Data Availability

Mendeley DataZEB Lab Measured Solar Radiation and PV Power Datasets (Original data).
